# Conservation machine learning

**DOI:** 10.1186/s13040-020-00220-z

**Published:** 2020-08-05

**Authors:** Moshe Sipper, Jason H. Moore

**Affiliations:** 1 Institute for Biomedical Informatics, University of Pennsylvania, Philadelphia, 19104-6021 PA USA; 2 Department of Computer Science, Ben-Gurion University, Beer Sheva, 8410501 Israel

## Editorial

Ensemble techniques—wherein a model is composed of multiple (possibly) weaker models—are prevalent nowadays within the field of machine learning (ML). Well-known methods such as bagging [1], boosting [2], and stacking [3] are ML mainstays, widely (and fruitfully) deployed on a daily basis. Generally speaking, there are two types of ensemble methods, the first generating models in sequence—e.g., AdaBoost [2]—the latter in a parallel manner—e.g., random forests [4] and evolutionary algorithms [5].

AdaBoost (Adaptive Boosting) is an ML meta-algorithm that is used in conjunction with other types of learning algorithms to improve performance. The output of so-called “weak learners” is combined into a weighted sum that represents the final output of the boosted classifier. Adaptivity is obtained by tweaking subsequent weak learners in favor of those instances misclassified by previous classifiers. The maximum number of estimators at which boosting is terminated is a free parameter that has to be carefully set by the user. The popular Scikit-learn Python package, used extensively within the ML community, sets this default value to 50 [6].

A random forest is an ensemble learning method that operates by constructing a multitude of decision trees at training time and then outputting the majority class (for classification problems) or mean prediction (for regression problems) of the individual trees. The number of trees is a free parameter set by the user; the default Scikit-learn value is 100 (up from 10 in past versions) [6].

An evolutionary algorithm is a population-based approach that inherently produces a cornucopia of models over generations of evolution. Most often one seeks a single, final model (or a Pareto set of models, when multiple objectives are sought). Yet, as eloquently suggested by [7] in their paper’s title, might we not obtain “Ensemble learning for free with evolutionary algorithms?” They proposed evolutionary ensemble learning, which extracts an ensemble either from the final population only or incrementally during evolution. Recently, [8] focused on genetic programming—wherein the individuals evolved are computational trees—introducing an ensemble coevolutionary algorithm that maintains two subpopulations, trees and forests, with the output model being a forest built as an ensemble of trees.

The number of models within an ensemble—the ensemble size—greatly impacts performance, yet there seems to be a dearth of studies addressing this issue. One recent theoretical study suggested that there is an ideal ensemble size under certain assumptions [9]. In practice, the ensemble size is set to some default value, or attempts are made to optimize this value either through a-priori, hyperparameter tuning or through online, dynamic sizing. And beyond ensemble approaches, any ML technique we employ is, in practice, run multiple times, producing a deluge of models.

We propose herein a different outlook altogether, seeking not an optimal ensemble size, but asking what might be accomplished if one is in possession of numerous models, either as an inherent part of the learning process, or simply due to many independent runs.

What we propose is simple in nature: Why not save—and possibly make use of—*all* the models? In a nod toward “save the trees” we designate this idea as *conservation machine learning*. Since we are expending considerable effort on producing models galore and evermore, why should we consign the vast majority of them to oblivion? Instead of considering most models as “failed” attempts along the way to a glorious winner, we look upon all as partial successes, possibly to be used beneficially at some future point.

Quite likely we shall end up with a large jungle of models rather than a small forest, a situation which may well require new thinking into the design of the ultimate answer to the problem at hand. In some cases, using classical approaches—e.g., majority voting (classification) or averaging (regression)—over the entire jungle might yield a good answer. In other cases we propose that the jungle could be cultivated, producing a garden of select models. Cultivation methods could be simple: select only models that meet certain criteria, or iteratively select models that improve performance; cultivation could also be more sophisticated, unleashing the full might of ML to produce meritorious gardens.

We are delving into new territory, advocating, as we were, sizeable ensembles. We believe this may well be advantageous where sizeable ensembles are generated as par for the course, a common-enough occurrence. After all, why waste a good—or even not-so-good—model?

We need not content ourselves to a per-run conservation approach, collecting only models from a single run. We can conserve models over multiple runs, and perhaps over multiple users. Consider current practice whereby cloud repositories store datasets, leaderboards, and—infrequently—a few choice models; why not store a jungle of models created by multiple users? Not only will this provide copious grist for the ML mill but, moreover, the cost of creating these models is often high—ML algorithms consume significant amounts of energy [10]—and limbo seems a somewhat unbecoming choice for their final resting place.

To drive this point home, think of the following scenario: Several research groups have been tackling an extremely hard problem (e.g., [11]), each group running variegated ML algorithms over several months (maybe years). It would not be hard to imagine that the number of models produced over time would run into the millions (quite easily more). Most of these models would be discarded unflinchingly, with only a minute handful retained, and possibly reported upon in the literature. The question we raise is: Could we produce better answers to the problem at hand if we had recourse to all the waste? For example, PennAI—an accessible AI system and open-source software—saves models over multiple runs (and possibly over multiple users), affording it the ability to glean insight into parameters from them all [12].

Using Scikit-learn it was quite straightforward to set up an exploratory experiment, with our choice of ML approach being the popular random forest.^1^ Through the make_classification function we generated 10 datasets, each comprising 1000 samples and a varying number of features, informative features, and classes. For each dataset we performed 30 replicate experiments, each with 5-fold cross validation. For each fold the dataset was split into a training set of 4 folds, and the left-out test fold. The training set was used in 100 independent runs to train a random forest of size 100. All trees across the 100 runs were saved into a jungle, whose size was 10,000 in the end. We then compared the performance of the forests vs. the jungle over the test set, and our results are shown in Table 1. While random forests do not necessarily attain high performance,^2^ conservation machine learning often shows significant improvement, demonstrating that the idea has at least *prima facie* merit. We fervently invite further exploration.

**Table 1:** Conservation random forests

Feat	Info	Cl	Perf forests	Perf jungles	Imp
10	3	2	0.85 (0.02)	0.85 (0.02)	0.0%
20	10	3	0.83 (0.02)	0.84 (0.02)	1.2%
50	40	4	0.64 (0.03)	0.69 (0.03)	7.8%
100	90	5	0.49 (0.03)	0.62 (0.03)	26.5%
200	150	6	0.3 (0.03)	0.45 (0.03)	50.0%
300	270	7	0.22 (0.03)	0.35 (0.03)	59.1%
400	350	8	0.18 (0.03)	0.29 (0.03)	61.1%
500	400	9	0.14 (0.02)	0.22 (0.03)	57.1%
1000	500	10	0.11 (0.02)	0.15 (0.03)	36.4%
1000	800	10	0.12 (0.02)	0.17 (0.03)	41.7%

Each line shows the results of 30 replicate experiments, with 5-fold cross validation, 100 independent runs per fold, forests of size 100, and resultant jungles of size 10,000. Feat: number of features in the dataset. Info: number of informative features. Cl: number of target classes. Perf forests: mean performance of forests on test set across all replicates (SD). Perf jungles: mean performance of jungles on test set across all replicates (SD); a jungle’s output was computed through straightforward majority voting. Imp: Percent improvement of Perf jungles vs. Perf forests

If one embraces a conservation approach to ML, other issues beyond those raised above will probably make the scene in short order. For example, of considerable interest nowadays are interpretability [13] and explainability [14] of AI-produced problem solvers. These issues are doubly important in the biomedical and healthcare fields, and will necessitate consideration under a conservation agenda [15].

It seems rather befitting to conclude with rapper and songwriter will.i.am who succinctly enunciated, “Waste is only waste if we waste it.”

## Data Availability

Not applicable.

## Abbreviations

AI: Artificial Intelligence
ML: Machine Learning

## Footnotes

1 Our code is available at https://github.com/EpistasisLab.

2 This was intended as an exploratory experiment; we used random forests and jungles in the most straightforward manner, with default settings, a fairly modest jungle size, and no garden cultivation.

## References

[1] Breiman L (1996). Bagging predictors. Mach Learn.

[2] Freund Y, Schapire RE (1995). A desicion-theoretica generalization of on-line learning and an application to boosting. European Conference on Computational Learning Theory.

[3] Wolpert DH (1992). Stacked generalization. Neural Netw.

[4] Ho TK (1995). Random decision forests. Proceedings of 3rd International Conference on Document Analysis and Recognition.

[5] Sipper M, Olson RS, Moore JH (2017). Evolutionary computation: the next major transition of artificial intelligence?. BioData Min.

[6] Pedregosa F, Varoquaux G, Gramfort A, Michel V, Thirion B, Grisel O, Blondel M, Prettenhofer P, Weiss R, Dubourg V, Vanderplas J, Passos A, Cournapeau D, Brucher M, Perrot M, Duchesnay E (2011). Scikit-learn: Machine learning in Python. J Mach Learn Res.

[7] Gagné C, Sebag M, Schoenauer M, Tomassini M (2007). Ensemble learning for free with evolutionary algorithms?. Proceedings of the 9th Annual Conference on Genetic and Evolutionary Computation.

[8] Rodrigues NM, Batista JE, Silva S (2020). Ensemble genetic programming. European Conference on Genetic Programming (Part of EvoStar).

[9] Bonab H, Can F (2019). Less is more: A comprehensive framework for the number of components of ensemble classifiers. IEEE Trans Neural Netw Learn Syst.

[10] García-Martín E, Rodrigues CF, Riley G, Grahn H (2019). Estimation of energy consumption in machine learning. J Parallel Distrib Comput.

[11] Bycroft C, Freeman C, Petkova D, Band G, Elliott LT, Sharp K, Motyer A, Vukcevic D, Delaneau O, O’Connell J (2018). The UK biobank resource with deep phenotyping and genomic data. Nature.

[12] Olson RS, Sipper M, La Cava W, Tartarone S, Vitale S, Fu W, Orzechowski P, Urbanowicz RJ, Holmes JH, Moore JH. A system for accessible artificial intelligence In: Banzhaf W, Olson R, Tozier W, Riolo R, editors. Cham: Springer: 2018. p. 121–34.

[13] Plumb G, Molitor D, Talwalkar AS (2018). Model agnostic supervised local explanations. Advances in Neural Information Processing Systems.

[14] Gunning D. Explainable artificial intelligence (XAI). Defense Advanced Research Projects Agency (DARPA), nd Web. 2017; 2.

[15] Hara S, Hayashi K, Storkey A, Perez-Cruz F (2018). Making Tree Ensembles Interpretable: A Bayesian Model Selection Approach. Proceedings of the Twenty-First International Conference on Artificial Intelligence and Statistics, vol. 84.

